# lop-DWI: A Novel Scheme for Pre-Processing of Diffusion-Weighted Images in the Gradient Direction Domain

**DOI:** 10.3389/fneur.2014.00290

**Published:** 2015-01-12

**Authors:** Farshid Sepehrband, Jeiran Choupan, Emmanuel Caruyer, Nyoman D. Kurniawan, Yaniv Gal, Quang M. Tieng, Katie L. McMahon, Viktor Vegh, David C. Reutens, Zhengyi Yang

**Affiliations:** ^1^Centre for Advanced Imaging, The University of Queensland, Brisbane, QLD, Australia; ^2^Queensland Brain Institute, The University of Queensland, Brisbane, QLD, Australia; ^3^Section of Biomedical Image Analysis, Department of Radiology, University of Pennsylvania, Philadelphia, PA, USA; ^4^School of Information Technology and Electrical Engineering, The University of Queensland, Brisbane, QLD, Australia

**Keywords:** spiral sampling, gradient direction domain, diffusion-weighted imaging, pre-processing, HARDI, local reconstruction

## Abstract

We describe and evaluate a pre-processing method based on a periodic spiral sampling of diffusion-gradient directions for high angular resolution diffusion magnetic resonance imaging. Our pre-processing method incorporates prior knowledge about the acquired diffusion-weighted signal, facilitating noise reduction. Periodic spiral sampling of gradient direction encodings results in an acquired signal in each voxel that is pseudo-periodic with characteristics that allow separation of low-frequency signal from high frequency noise. Consequently, it enhances local reconstruction of the orientation distribution function used to define fiber tracks in the brain. Denoising with periodic spiral sampling was tested using synthetic data and *in vivo* human brain images. The level of improvement in signal-to-noise ratio and in the accuracy of local reconstruction of fiber tracks was significantly improved using our method.

## Introduction

Diffusion-weighted imaging (DWI) is a powerful tool for inferring tissue structure and has been used extensively to map white matter pathways in healthy and diseased brains (1, 2). Errors due to thermal and physiological noise and to eddy currents affect individual diffusion-weighted images and influence the accuracy of measures obtained from DWI data such as fractional anisotropy, fiber orientation, and separation between fibers (3–5).

Averaging over a number of data acquisitions improves the signal-to-noise ratio (SNR) but is time consuming, limiting practicality. Hence, several post-processing techniques have been proposed to enhance the quality of diffusion-weighted images. Parametric and non-parametric statistical approaches have been used to describe the noise distribution and to derive the best fit for tensor parameters (6–8). Parametric methods rely on the distributional model of noise being correct. Non-parametric statistical approaches are model independent but are less powerful in hypothesis testing and more computationally demanding (7).

A number of techniques that do not require statistical analysis have been employed to enhance information contained in diffusion-weighted images (7). Image SNR can be enhanced by filtering individual images using the wavelet shrinkage technique (9). This approach is highly efficient due to the sparse representation; however, early implementations assumed spatially homogeneous variance of noise. Nowak and Pajevic described a method of wavelet-based denoising with spatially inhomogeneous noise but this approach suffered from the disadvantage of blurring edges in the image (7, 10). SNR can also be improved by exploiting spatial variations and similarities across an image, for example, using denoising techniques such as non-linear anisotropic diffusion, non-local means, or their variants (11–19). Alternatively, regularization functions can be used in the model fitting stage to increase the accuracy of parameter extraction (20, 21).

Incorporation of prior knowledge may also increase the effectiveness of denoising. In this paper, we propose a sampling and pre-processing scheme in which gradient direction encodings vary in continuous steps on a unit sphere, resulting in a periodic diffusion-weighted signal in each voxel. In this domain, which we call the “gradient direction domain,” the diffusion signal in each voxel is concentrated in the low-frequency portion of the Fourier spectrum of the acquired signal. This prior knowledge can be used for effective filtering of signal from noise using simple low-pass filtering in the Fourier domain.

To assess the impact of our method on the accuracy of diffusion-weighted data analysis, we first evaluated it on a digital phantom, where ground truth signal and fiber orientations are known. We compared mean squared error (MSE) before and after pre-processing datasets across a wide range of SNR levels. Local reconstruction accuracy was evaluated according to fiber population estimation (number of fiber bundles in each voxel) and angular estimation. For human brain data, we concatenated eight DWI acquisitions in the same subject to provide “gold standard” data similar to (22). The accuracy of the new method was assessed in terms of SNR improvement and accuracy of fiber population estimation.

## Method

### Pre-processing with low-pass DWI

**Figure 1:**
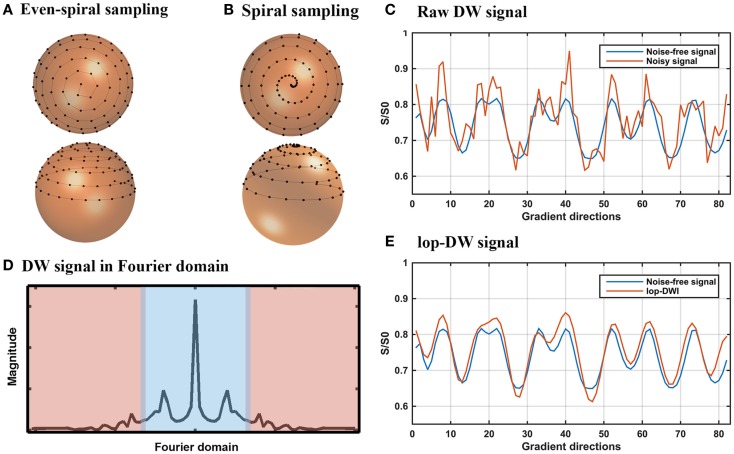
**lop-DWI: sampling and filtering**. **(A)** Evenly distributed spiral samples over the unit sphere, **(B)** periodic spiral sampling over the unit sphere, sampled with 20° azimuthal angular velocity, **(C)** noise-free and noisy diffusion-weighted signals of a given voxel of the phantom in the gradient direction domain, **(D)** a representative view of diffusion-weighted signal in frequency domains, where blue area represents low-frequency part of the diffusion-weighted signal, **(E)** noise-free and filtered diffusion-weighted signals of a given voxel of the phantom in the gradient direction domain.

A periodic and continuous arrangement of gradient directions according to orientation results in periodicity of the diffusion-weighted signal in each voxel, which can be exploited to denoise DWI data using frequency domain techniques in a voxel-wise manner. Gradient directions used in DWI can be mapped to points on a unit sphere, which are parameterized by the longitude angle and latitude angle. Here, we term this parameterization the “gradient direction domain.” A spherical spiral curve can be defined to traverse gradient direction domain, forming a one-dimensional continuous indexing of gradient directions (Figure 1C). This one-dimensional indexing is discretized to generate a sample set of gradient directions **d**_n_. The acquired diffusion-weighted signals in each voxel form a one-dimensional signal, **s**, in the gradient direction domain, which is defined as follows:

(1) $$\begin{array}{lll} \text{s} & = & \left[s_{1},\ldots ,s_{N}\right],\\ s_{n} & = & E\left(b,{\text{d}}_{n}\right)∕E_{0},\\ b & = & \left(\Delta -\delta ∕3\right){\left(\delta \gamma \left|G\right|\right)}^{2}, \end{array}$$

where *E*(*b*,**d**_n_) is the diffusion-weighted signal when the gradient is applied in direction **d**_n_, *b* (the *b-*value) is an example for a Stejskal–Tanner pair of diffusion gradients (23), *E*_0_ is the signal without diffusion weighting (*b* = 0), Δ is the diffusion time, δ is the pulse length, γ is the gyromagnetic ratio, and *G* is the gradient strength. The periodic and continuous samples yield a signal **s** that is pseudo-periodic, continuous, and “smooth” (i.e., *s*_n_ ≈*s*_n+1_) and that concentrates at low frequencies (Figure 1D).

In our previous work, we outlined a spiral sampling scheme (24) as shown in Figure 1B. Such a spiral sampling scheme can be used to improve reconstruction of local extrema using techniques such as low-pass filtering but the samples are unevenly distributed over the sphere, potentially leading to orientation-dependent bias (25, 26). Uniform distribution of periodic spiral samples over the unit sphere avoids orientation-dependent bias (25). Thus, we used the sampling scheme proposed by Koay (27), which yields uniformly distributed periodic spiral samples based on an analytically exact spiral over the sphere (Figure 1A). Since the acquired DWI data using this sampling scheme retain intrinsic periodicity and “smoothness,” low-pass filtering can be applied to remove noise. To this end, **s** is detrended by subtracting its least squares linear fit, then low-pass filtered by keeping low-frequency components in the Fourier domain and zero filling the rest (Figure 1E). To simplify the wording, we term DWI using spiral sampling combined with subsequent low-pass filtering “low-pass DWI (lop-DWI).” We applied lop-DWI on datasets acquired with uneven- and even-spiral sampling schemes, passing approximately 25% of low-frequency components in the Fourier domain (cut-off value of 11). This cut-off value was chosen by comparing results of all possible cut-off values based on the local reconstruction accuracy, of which the details will be given in Section “Evaluation of Digital Phantom Data.”

To compare the efficacy of our method, multiple DWI data sets were generated on a digital phantom and acquired on a human volunteer using three different sampling schemes:

(1) Even sampling: eighty-two uniformly distributed samples on a hemisphere obtained using the electrostatic repulsion technique (25);
(2) Even-spiral sampling: uniformly distributed samples based on an analytically exact spiral (27) (Figure 1A). To generate a uniform distribution of samples satisfying the criterion of antipodal symmetry, 164 samples were generated on a unit sphere and 82 of the points that were located on a hemisphere were selected, and arranged as shown in Figure 1A;
(3) Uneven-spiral sampling with periodic angular steps (24). An azimuth angular step of 20**°** was used to generate 82 directions on a hemisphere, using an in-house script, written in MATLAB (Mathworks, Natick, MA, USA).

### Generation of digital phantom data

**Figure 2:**
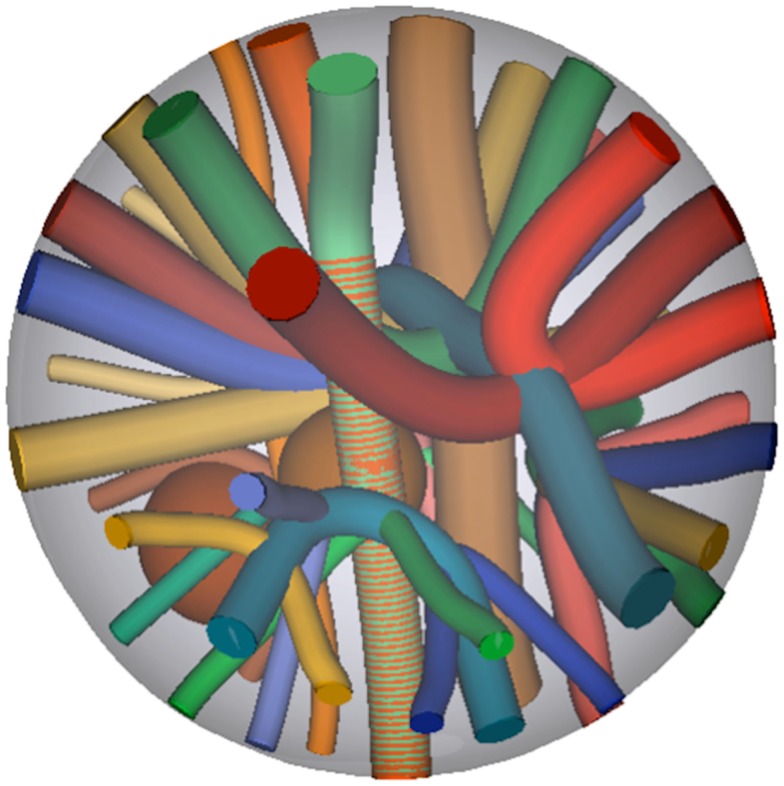
**Ground truth fiber bundle geometries of the phantom, from which Rician noise-corrupted DWI were simulated**.

Digital phantom data were generated using *b*-values of 1,000 and 3,000 s/mm^2^. To test the performance of each scheme in denoising and local reconstruction, Rician noise was added to simulate SNR ranging from 10 to 100 in steps of 10. SNR of 5, 125, and 150 were also included as extreme cases. The phantom consists of a set of 27 fiber bundles contained within a spherical domain as depicted in Figure 2, from which DWI were simulated using Phantomas1. The signal in white matter simulates both intra- and extra-axonal diffusion, using a model similar to CHARMED (28).

### Evaluation of digital phantom data

To estimate the amount of improvement in the signal, the MSE of each dataset of different SNRs was calculated taking the ground truth signal as reference. The mean and SD of MSE across all the voxels excluding background were computed for each case.

We compared the effects of our method on the accuracy of local reconstruction of fiber orientations with regularized Q-Ball Imaging (QBI) (20) and Constrained Spherical Deconvolution (CSD) (29). QBI was chosen because it also involves spherical low-pass filtering. A regularization constant of 0.006 was used as suggested in (20, 22, 26, 30). Dipy software (31) was used to estimate the Fiber Orientation Distribution (FOD) in each voxel using a spherical harmonic of order 8 and to identify the peaks of the FODs. It was assumed that each voxel contained a maximum of three fibers. We also applied the CSD method to the phantom data, as this technique was used to evaluate human brain data. We generated phantom data with the same SNR measured from the human brain data that we collected and applied CSD technique with suggested parameter settings from literature (22, 30), on both DWI and lop-DWI data. In what follows, we call QBI and CSD reconstruction of lop-DWI data lop-QBI and lop-CSD, respectively.

Two metrics were used to compare the results of local reconstruction between methods (32):

(1) Angular error of fiber bundle reconstruction. The angular error of estimated fiber bundle orientation was defined as the angular difference (in degrees) between the ground truth peaks and the closest estimated peaks in a voxel:
  (2) $$\mathrm{Angular}\;\mathrm{error}=\frac{\pi }{180}\;\arccos\left(\left|{\text{p}}_{\mathrm{gt}}\cdot {\text{p}}_{i}\right|\right),$$
  where **p***_gt_* and **p***_i_* are ground truth peak and an estimated peak of interest in each voxel, respectively. For a voxel containing multiple fiber bundles, the average angular error across fiber bundles was taken as the voxel-wise angular error.
(2) Error in the estimated number of fibers. The number of peaks in the FODs was counted as the number of fibers. Estimation error was then calculated by comparing the estimated number of fibers with ground truth. The percentage of voxels with over- or under-estimation of fiber number and the success rate, defined as the percentage of voxels in which all fibers were successfully reconstructed, are reported. Statistically significant differences (*p* < 0.05) in the two metrics between lop-QBI data and QBI data were identified using paired *t*-test.

### Data acquisition in a human subject

The medical research ethics committee of the University of Queensland approved the study, under the guidelines of the National Health and Medical Research Council of Australia. Informed consent was obtained from the volunteer. The image dataset was anonymized. A healthy volunteer was scanned on a Siemens 3 T Trio using a 32-channel head coil over a period of 3 h to acquire 12 sets of DWI data with the following sampling schemes: eight datasets with electrostatic even sampling (25), optimally ordered as described in Ref. (33); two datasets with even-spiral sampling (Figure 1A); and two datasets with uneven-spiral sampling (Figure 1B). For each dataset, 82 diffusion-weighted images with at least three unweighted (*b*_0_) images were acquired. A twice-refocused bipolar diffusion spin-echo sequence (34) was used with 2.5 mm isotropic resolution, TE/TR = 112/9400 ms, partial Fourier filling of 6/8, matrix size of 100 × 100 × 55, *b* = 3,000 s/mm^2^ (26), iPAT = 2 with GRAPPA reconstruction. The acquisition time to obtain each dataset was ~14 min.

The eight datasets with electrostatic even sampling were concatenated (not averaged) into a single dataset, resulting in a total of 656 DWIs and 48 *b*0s (The eight datasets have been made available at https://sites.google.com/site/lopdwi). Images were corrected for subject motion and residual eddy current-induced geometric distortions with the required *B*-matrix adjustments using ExploreDTI software (35). Lop-DWI data were obtained from datasets acquired using even (“even lop-DWI”) and uneven (“uneven lop-DWI”) spiral schemes using a cut-off frequency of 11.

### Evaluation of human brain data

The impact of lop-DWI was assessed in the human brain data by comparing SNR and the accuracy of estimates of fiber number. The corpus callosum was selected as the white matter structure of interest. It was segmented by choosing voxels with FA >0.7 and a principal eigenvector in the *x*-direction. SNR was calculated (31) by dividing the mean signal in the region of interest by the SD in background (36) voxels identified using median Otsu segmentation (31). For each dataset, we measured SNR across all gradient directions and the average value is reported here. The results from the eight evenly sampled datasets were averaged and are also included in the SNR comparison.

Local reconstruction accuracy was evaluated by comparing the estimation accuracy of fiber number across white matter regions. We used the CSD technique with suggested parameter settings in (22, 30). MRtrix (30) was used to apply CSD on all of the evenly sampled DWI data and lop-DWI data. Similar to phantom data, spherical harmonic order up to degree 8 was used to obtain FODs. For each dataset, voxels with FA >0.7 were identified and the spherical harmonic decompositions of all the resulting profiles were then averaged to estimate the response function. Fibers of each voxel were extracted by applying a threshold of 0.1 to FOD peak amplitude, assuming maximum fiber population of three for each voxel.

From the peaks, we calculated the percentage of voxels in white matter with single, two, and three (or more) fibers. To obtain region-specific statistics on fiber population, the Harvard–Oxford subcortical structural atlas (37) was used to segment out the white matter and the JHU–MNI–ss atlas (38) was used to segment white matter into 45 sub-regions. All masks were then eroded by one voxel to exclude boundary voxels with uncertainty. Masks were obtained using DSIStudio software (39).

## Results

### Phantom data

#### Signal-to-noise ratio

**Figure 3:**
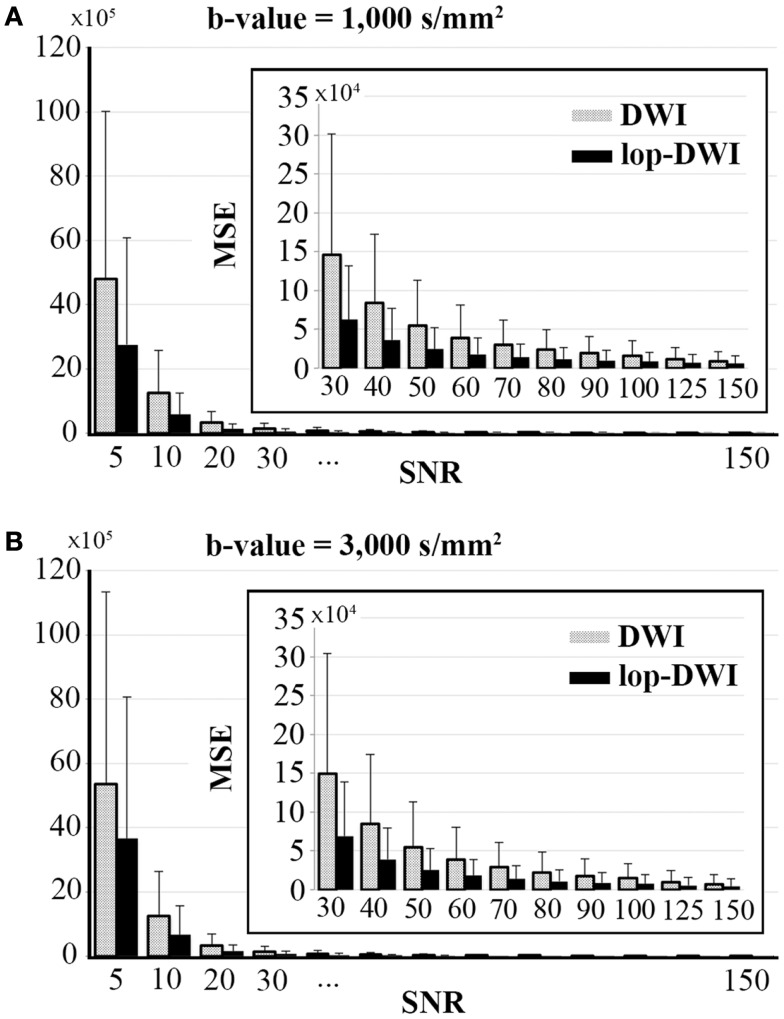
**Mean square error over different SNRs for digital phantoms, simulated with (A) *b*-value = 1,000 s/mm^2^, and (B) *b*-value = 3,000 s/mm^2^**. To aid visualization of MSE values, bar charts for SNRs above 30 were expanded and shown inside each plot.

Voxel-level MSE comparison across different datasets showed that mean MSE is significantly decreased when lop-DWI is applied (*p* < 1e−10), as summarized in Figure 3. For *b*-value = 1,000 s/mm^2^, MSE of even lop-DWI data with SNR = 20 were lower than that from DWI with SNR = 30. For *b*-value = 3,000 s/mm^2^, MSE of even lop-DWI data with SNR = 20 was only slightly higher than that from DWI with SNR = 30. For both *b*-values, MSE of lop-DWI for phantom data with SNR = 30 were as low as those from DWI data with SNR = 50. Note that even for SNR value as high as 150, significant improvement in signal was obtained (*p* < 1e−10, paired *t*-test). MSE of even lop-DWI and uneven lop-DWI data were almost equal (data are not shown here).

#### Angular accuracy

**Figure 4:**
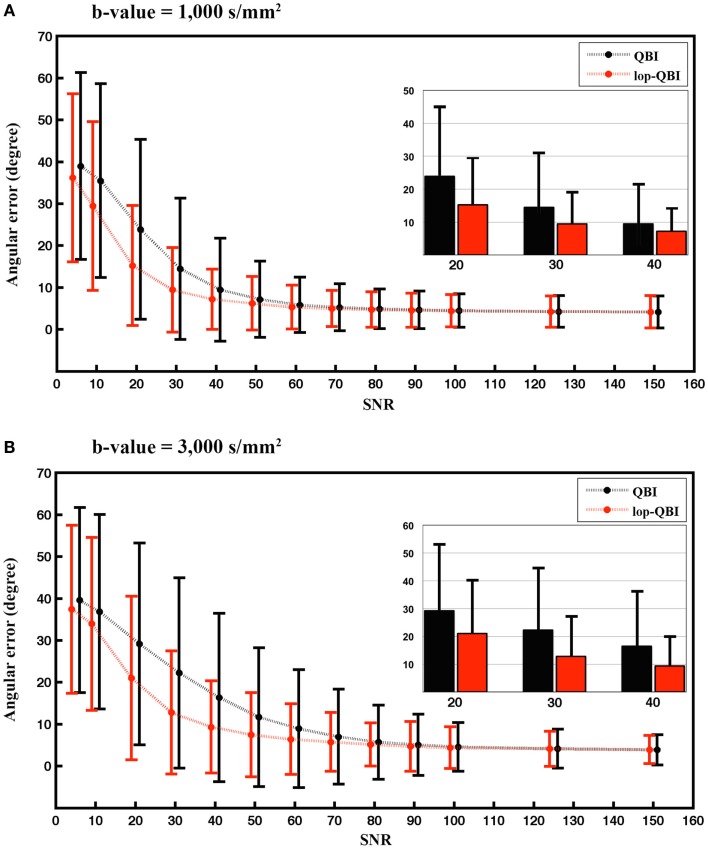
**Mean and SD of angular error between fibers of digital phantom and the ground truth, measured from raw-DWI (black) and lop-DWI (red), across different SNRs**. **(A)** is the results from simulation with *b*-value = 1,000 s/mm^2^ and **(B)** with *b*-value = 3,000 s/mm^2^. To aid visualization, mean and SD of angular error for SNRs of 20, 30, and 40 are illustrated separately inside each plot. In **(A)**, all the mean differences ≤50 are statistically significant. In **(B)**, all the mean differences ≤70 are statistically significant (*p* < 1e−10).

Figure 4 shows that angular accuracy was improved when using lop-QBI, particularly when SNR was low. For *b*-value = 1,000 s/mm^2^, decreases of angular error were significant for SNRs smaller than or equal to 50 (*p* < 1e−10). This held for SNRs smaller than or equal to 70, when *b*-value = 3,000 s/mm^2^ was used (*p* < 1e−10). For high SNRs, improvements in angular estimation were slight or unnoticeable. However, for low SNRs, such as SNR = 20, the mean angular estimation was improved by almost 10°.

#### Fiber population

**Figure 5:**
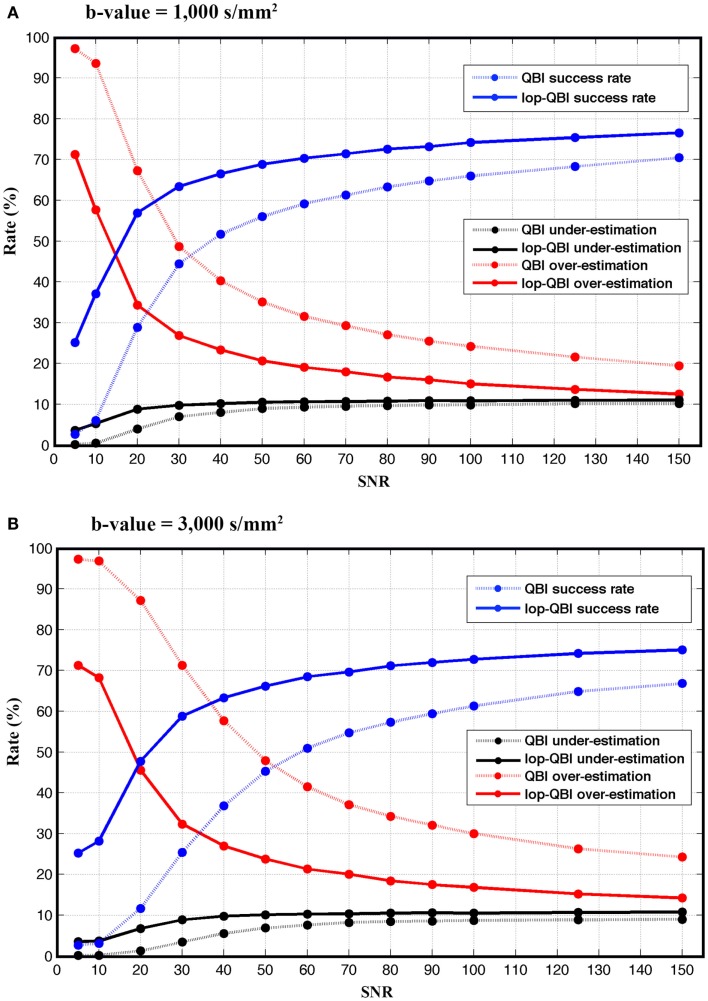
**Success, over-estimation, and under-estimation rates (see Method), measured from raw-DWI (dotted lines) and lop-DWI, across different SNRs**. **(A)** is the results from simulation with *b*-value = 1,000 s/mm^2^ and **(B)** with *b*-value = 3,000 s/mm^2^.

Figure 5 shows that the achieved success rate from lop-QBI was notably higher than that from QBI. For instance, for both *b*-values, success rate of lop-QBI when SNR = 50 were higher than or as high as the highest QBI success rate. For *b*-value = 3,000 s/mm^2^ and for SNR = 20 and 30, success rate increased almost 35%, which can be explained by the substantial decrease in the over-estimation rate. For low SNR, spurious fibers were reconstructed almost in all the voxels, which were the consequence of fitting the model to the noise. This explains low under-estimation rate when SNR was low. Over- and under-estimation rate of lop-QBI were almost equal when SNR was high. However, QBI over-estimation rates were always higher than under-estimation. Under-estimation rate never exceeded 11% across all datasets. Under-estimation rate of lop-QBI were slightly higher than QBI, and reached similar value as SNR increased. Observed under-estimation rates for high SNR came from voxels with low fiber crossing angle. It is known that high angular resolution diffusion imaging (HARDI) techniques cannot resolve low angles. We note that under-estimation rates were very low when SNR was low (<30). This is because, when SNR is too low, high fluctuation in diffusion-weighted signal leads to reconstruction of spurious fibers, causing almost no under-estimation and high over-estimation.

**Figure 6:**
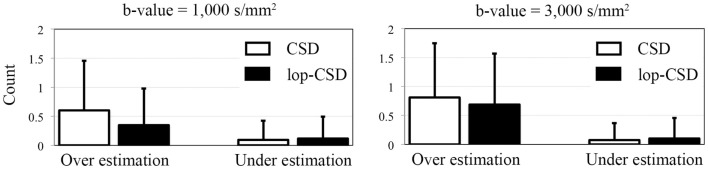
**Mean and SD of error in the estimation of number of fibers**. Over-estimation errors of lop-CSD were significantly lower than that obtained from CSD (*p* < 0.01). Both CSD and lop-CSD had low under-estimation values, while lop-CSD had insignificantly higher under-estimation.

When these local reconstruction analyses were performed on phantom data with SNR 40 (similar to our human data) using CSD, similar pattern was observed in the estimation of number of fiber bundles (Figure 6). However, for CSD, no significant improvement in angular estimation was apparent (not shown here).

#### Even sampling vs. uneven sampling

**Figure 7:**
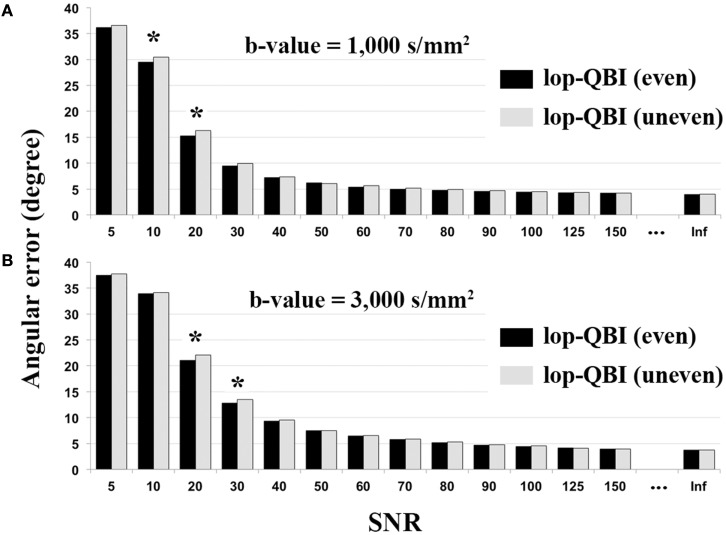
**Mean of angular error between fibers of digital phantom and the ground truth, measured from evenly sampled lop-DWI (Figure 1A) and unevenly sampled lop-DWI (Figure 1B), across different SNRs**. **(A)** is the results from simulation with *b*-value = 1,000 s/mm^2^ and **(B)** with *b*-value = 3,000 s/mm^2^. Asterisks show statistically significant difference in mean values (*p* < 0.05).

Figure 7 shows that evenly sampled lop-QBI data had slightly higher SNR than unevenly sampled lop-QBI data. The differences in the mean angular error between the two techniques were significant only in four datasets (see asterisks in Figure 7). It should be noted that unevenly sampled lop-DWI outperformed DWI in all the above criteria, but the improvement was slightly weaker than evenly sampled lop-DWI. Moreover, results (not shown here) from raw data acquired with evenly sampled data and with evenly sampled spiral data were almost the same but slightly different from that with unevenly sampled spiral data, which confirms the importance of even distribution of sampling to avoid orientation variance in the distribution of noise (26).

### Human data

#### Signal-to-noise ratio

Table 1 indicates that SNR of diffusion-weighted images was improved significantly in the evenly sampled lop-DWI results. SNR was improved almost 28% using our approach. SNR of lop-DWI for both evenly and unevenly sampled spiral data was significantly higher than raw images (*p* < 1e−10). In addition, the results of paired *t*-test indicated that SNR of lop-DWI of evenly sampled spiral data was significantly higher than SNR of all of eight DWI repeats (*p* < 0.05). SNR of first eight scans was significantly higher than SNR of all raw images. Surprisingly, it was not significantly higher than any of the results of lop-DWI for evenly sampled spiral data (*p* ≈ 0.2). It should be noted that SNR of raw images from unevenly sampled spiral data were significantly lower than other raw images. This is because in this scheme, the samples are unevenly distributed over the sphere, which leads to an uneven propagation of noise. SNR of each image varies depending on the gradient direction and the region of interest. Spiral sampling has dense acquisition along one axis and coarse acquisition along the others. Therefore, the estimated mean SNR will be weighted by the SNRs of the region with the dense acquisition. In our experiment, more data were acquired in the direction that has low SNR (*x*-direction).

**Table 1:** **Mean and SD of SNR across all gradient directions of raw-DWIs and lop-DWIs of human brain data, obtained from different sampling schemes**.

Sampling	Scan repeat	Raw-DWI^a^		Lop-DWI	
		Mean SNR	SD	Mean SNR	SD
Even	1st	39.6	22.9	–	–
	2nd	39.7	23.2	–	–
	3rd	39.9	23.4	–	–
	4th	38.8	23.1	–	–
	5th	39.9	23.7	–	–
	6th	39.2	23.0	–	–
	7th	39.8	23.2	–	–
	8th	39.1	23.2	–	–
Even-spiral	1st	39.2	23.1	**48.5**^b^	26.8
	2nd	38.6	22.8	**49.3**^b^	27.4
Spiral	1st	31.4	23.7	38.4	29.5
	2nd	31.7	24.2	38.5	29.7
Average of eight evens	–	**54.9**^b^	31.8	–	–

*Datasets are listed in the same order as they were scanned*.

*^a^Raw-DWI were corrected for motion, eddy current, and EPI distortions (see Method)*.

*^b^Indicates significant difference, using paired *t*-test (see text)*.

**Figure 8:**
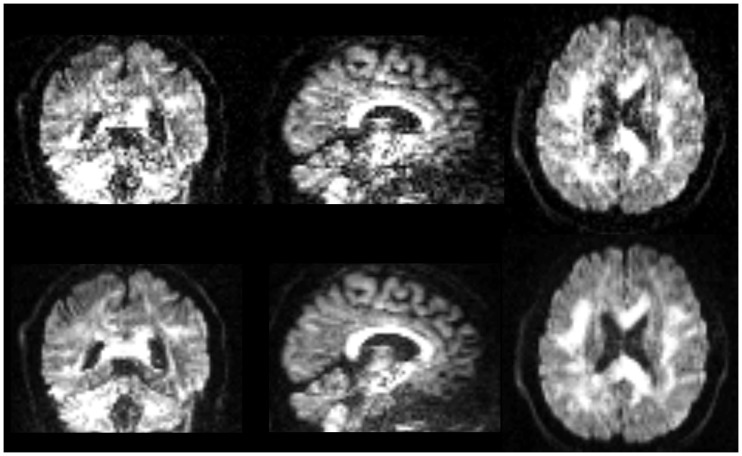
**Images are instances of human brain DWI from a given gradient direction, from raw-DWI (first row) and lop-DWI (second row)**. Raw-DWI was corrected for motion, eddy current, and EPI distortion (see Method).

Qualitative comparison of lop-DWI based on a specific diffusion-gradient direction and raw images after correction for motion, eddy currents, and EPI distortions shows that quality of DWI was improved (Figure 8). Note that the improvement can be appreciated in the spatial domain, although lop-DWI was applied at voxel level in the gradient direction domain.

### Fiber population

**Figure 9:**
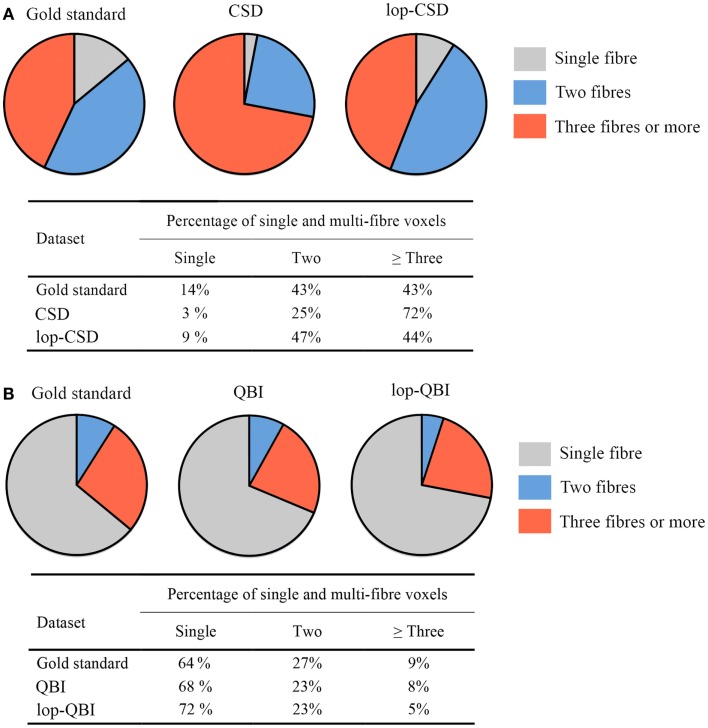
**Fiber population count across voxels of the white matter, (A,B) percentages of voxels with single, two, or greater than three fiber populations across the whole white matter, estimated for gold standard (see Method), raw–DWI, and lop-DWI, using (A) CSD, and (B) QBI techniques**.

When CSD was used, fiber bundle counts in lop-DWI data were notably similar to that obtained from the concatenation of eight DWI datasets (Figure 9A). Percentage of voxels with three fibers (or more) was much smaller in lop-DWI than DWI, but close to the ground truth, showing low over-estimation rate with the use of lop-DWI. Our results agree with those published by Jeurissen et al. (22). It should be noted that, same values were obtained from each one of eight repeats of the DWI data, or from each one of two repeats of lop-DWI. Therefore, only one pie chart was drawn for each method.

Across all datasets processed using QBI (Figure 9B), large percentages of voxels were estimated to have only single fiber (more than 60%), which is in contrast with the study of Jeurissen et al. (22). This suggests that CSD is more powerful in extracting information on the nature of fiber bundles in voxels.

**Figure 10:**
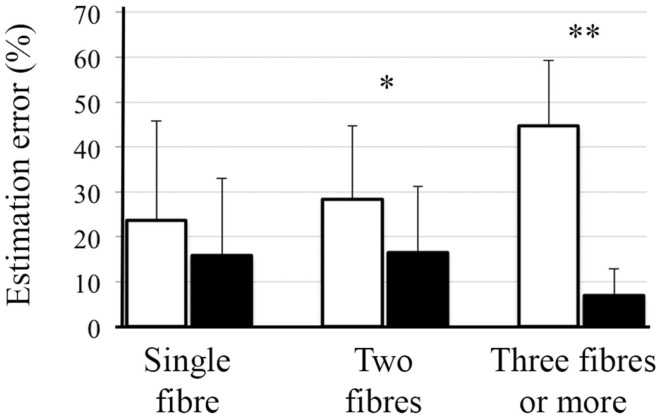
**Mean and SD of error in estimation of number of fibers, using CSD (white bars) and lop-CSD (black bars), compare with estimated values from gold standard data (see Method)**. * and ** indicate significant levels of *p* < 0.01 and *p* < 1e−10, respectively. Paired *t*-test was used.

Region-specific fiber population estimates using CSD are reported in Table 2. Estimated values indicate that lop-DWI results were considerably closer to gold standard compared with those obtained from DWI. In different regions of corpus callosum, such as Tapetum, Genu, and Splenium, for the gold standard and lop-DWI cases, majority of voxels had single fiber. However, over-estimation was observed in the majority of voxels when raw images were used. The same trend held for other regions too. For example, most of the voxels of corticospinal tracts were estimated to have two fibers for the gold standard and lop-DWI cases, while most of the voxels of the raw-DWI (third column) were estimated to have three fibers. The mean error in estimation of number of fibers using lop-CSD was significantly lower than that using CSD (Figure 10). Mean difference between gold standard and lop-CSD in voxels with single fiber was insignificant (*p* = 0.06). However, for voxels with multi-fiber population, errors of estimation of lop-CSD were significantly lower than DWI.

**Table 2:** **Percentages of voxels with single, two, or greater than three fiber populations across regions of white matter, estimated for gold standard (see Method), DWI, and lop-DWI (extended from Figure 9)**.

The regions of interest	Gold standard			Lop-DWI			DWI		
	1	2	≥3	1	2	≥3	1	2	≥3
Pontine crossing tract	0	57	42	0	53	46	0	9	89
Genu CC	73	22	3	61	32	6	11	33	55
Splenium CC	81	16	2	59	37	3	18	50	30
Body CC	69	26	4	35	58	5	16	49	34
Tapetum_R	92	7	0	66	33	0	34	46.	18
Tapetum_L	95	4	0	48	52	0	21	53	25
Fornix – column and body	37	7	5	18	72	8	5	20	74
Corticospinal tract R	1	30	18	0	78	21	0	14	85
Corticospinal tract L	7	77	14	1	74	24	0	18	81
Medial lemniscus R	0	56	43	0	83	16	0	11	88
Medial lenuiiscus L	0	61	38	0	84	16	0	13	86
Inferior_cerebellar_peduncle_R	13	42	44	0	48	51	0	11	88
Inferior_cerebellar_peduncle_L	9	53	37	3	59	37	0	15	84
Superior_cerebellar_peduncle_R	52	35	11	12	69	17	3	14	82
Superior_cerebellar_peduncle_L	52	42	5	5	72	22	1	13	85
Cerebral_peduncle_R	54	32	12	1	72	26	4	22	72
Cerebral_peduncle_L	55	34	9	1	81	16	4	27	67
Anterior_limb_of_internal_capsule_R	16	43	40	16	59	24	1	12	86
Anterior_limb_of_internal_capsule_L	11	56	32	13	69	17	0	25	75
Posterior_limb_of_internal_capsule_R	42	43	14	5	78	15	3	40	56
Posterior_limb_of_internal_capsule_L	36	51	12	2	78	18	5	47	47
Retrolenticular_part_of_internal_capsule_R	22	60	16	13	66	19	4	43	52
Retrolenticular_part_of_internal_capsule_L	23	64	11	32	56	11	2	43	53
Anterior_corona_radiata_R	6	57	35	3	49	47	0	21	78
Anterior_corona_radiata_L	4	55	39	5	60	34	1	18	80
Superior_corona_radiata_R	8	70	20	1	79	19	1	25	73
Superior_corona_radiata_L	7	70	21	0	83	16	0	33	65
Posterior_corona_radiata_R	12	77	10	4	81	13	0	43	51
Posterior_corona_radiata_L	6	73	19	3	66	29	3	30	65
Posterior_thalamic_radiation_R	40	53	5	23	66	10	9	49	41
Posterior_thalamic_radiation_L	44	50	4	24	70	4	10	54	34
Sagittal_stratum_R	30	60	8	26	60	12	4	47	48
Sagittal_stratum_L	23	64	11	32	44	24	0	38	60
External_capsule_R	8	55	35	3	65	31	0	28	71
External_capsule_L	15	57	26	3	64	31	0	27	72
Cingulum_(cingulate_gyrus)_R	22	63	9	24	56	18	1	44	53
Cingulum_(cingulate_gyrus)_L	20	64	14	31	48	20	0	45	53
Cingulum_(hippocampus)_R	17	24	58	8	28	63	0	7	92
Cingulum_(hippocampus)_L	23	43	28	15	45	38	0	9	90
Superior_longitudinal_fasciculus_R	1	57	41	12	55	32	0	34	65
Superior_longitudinal_fasciculus_L	2	52	45	9	52	37	0	41	58
Superior_frontooccipital_fasciculus_R	12	45	41	0	53	46	0	17	82
Superior_frontooccipital_fasciculus_L	0	45	54	0	31	68	0	0	100
Uncinate_fasciculus_R	23	69	7	0	100	0	0	66	33
Uncinate_fasciculus_L	13	86	0	0	93	6	0	25	75

## Discussion

We outlined a new pre-processing technique to enhance diffusion-weighted data. The technique is based on spiral sampling of diffusion-weighted images in gradient direction domain that results in periodicity in the acquired signal in each voxel. In the acquired signal, fiber information concentrates in the low frequencies of the Fourier spectrum, and noise in high-frequencies. The analytically exact spiral scheme of Koay (27) was used to obtain evenly distributed, yet spiral samples over the sphere. The low-frequency part of the diffusion-weighted signal, when acquired in the gradient direction domain, contains information on the orientation of underlying fibers. Therefore, filtering in this domain removes noise and increases accuracy of fiber reconstruction (i.e., a lower rate of spurious fiber reconstruction).

Using a digital phantom and *in vivo* human brain imaging data, we showed that the spiral sampling-based filtering scheme significantly improves SNR, increasing the accuracy of fiber reconstruction from diffusion-weighted brain imaging data. SNR obtained from human brain data filtered using our method improves the DWI quality by as much as 25% (Table 1). SNR of filtered data (~50) was close to the SNR of the data obtained from average of eight scans (~55). It should be noted that SNR improvement with the lop-DWI method was achieved in gradient direction domain, and not with spatial filtering. This may lead to over smoothing of regions with high spatial variation, i.e., blurring of edges.

Local reconstruction of lop-DWI (evenly distributed spiral sampling scheme with low-pass filtering) was more accurate compared with some conventional techniques. In particular, over-estimation rate was significantly lower with the lop-DWI approach. Figures 9 and 10 show that estimated fiber population values in different regions of white matter, obtained from lop-DWI, were very close to that obtained from our gold standard data and, to that obtained in a similar experiment by Jeurissen et al. (22). Accurate estimation of the number of fiber bundles provides additional information for interpretation of structural changes of underlying tissue and increase specificity of DTI analysis. For example, it is possible to assess if an increase or decrease in FA value is due to the loss of one of the fibers. Moreover, robust estimation of number of fiber bundles is practical for HARDI techniques that aim to quantify structural characteristics of each fiber bundle of interest separately, one of which is the apparent fiber density technique (40).

To apply a voxel-wise signal processing technique such as lop-DWI to data acquired with conventional schemes, uniform samples can be rearranged in the gradient direction domain and smoothing techniques applied. Regardless of the sampling scheme used, the diffusion-weighted signals in a given voxel form a 1D signal in the gradient direction domain. By re-arranging the 1D signal in this domain, one can benefit from prior knowledge about the signal and noise. For example, when the diffusion-weighted signals corresponding to proximal gradient directions are arranged so that neighboring directions are adjacent to each other, the 1D signal is smooth, allowing smoothing techniques such as wavelet denoising or quadratic smoothing to be applied. lop-DWI uses a periodic spiral scheme leading to a smooth and periodic signal in each voxel. This prior knowledge allows Fourier-based smoothing, which we found to be more effective than other smoothing techniques (the comparison of different denoising techniques is not reported in this manuscript).

Here, we used a digital phantom with a comprehensive representation of white matter microstructure model and additive Rician noise. While the phantom takes into consideration a variety of tissue characteristics, it does not represent the entire complexity of brain microarchitecture. For example, the phantom does not take into account spin exchange among intra-cellular and extra-cellular spaces, which occurs due to membrane permeability (41). An exact parametric representation of the highly complex structure of human brain is impossible. In this study, we used an existing model that has been previously tested in the literature to generate the phantom (28) and the widely accepted Rician noise distribution (42, 43).

For the phantom study, where ground truth signal was available, we assessed the amount of signal improvement by estimating MSE between the filtered datasets and the ground truth. Results showed that significant improvement could be obtained with lop-DWI (Figure 3). For *in vivo* human brain imaging, due to the large number of contributing factors, exact estimation of SNR is challenging. In DWI, SNR depends not only on the region of interest but also on the direction of the applied diffusion gradient. Therefore, we calculated SNR across all diffusion gradient directions using the same region of interest (corpus callosum) for all of the acquired datasets and reported the mean and SD. This analysis approach also provided the opportunity to test for significant changes in the means.

Regularized QBI and CSD were chosen for fiber reconstruction. QBI utilizes spherical low-pass filtering, which enables us to compare our method on one of the state-of-the-art techniques that involves low-pass filtering. CSD was chosen to replicate similar experiment (22) for human brain data to justify our gold standard values.

In the phantom, ground truth peaks of FOD were available, which enabled voxel-level assessment of local reconstruction accuracy of different techniques. For human data with the absence of ground truth, we created a gold standard by concatenating eight repeats of DWIs. Local reconstruction of the concatenated data was used as gold standard to assess the local reconstruction accuracy of each technique. In *in vivo* experiments, we observed that voxel-level comparison might be biased due to misalignment between datasets and motion, even after motion correction. Therefore, region-specific statistics were calculated for evaluation. These considerations for the *in vivo* experiment make it challenging to assess particular local reconstruction criteria, such as angular error. Therefore, a similar approach to that proposed by Jeurissen et al. (22) was adopted to assess local reconstruction accuracy of the competing techniques. Although some inferences can be made on the basis of concordance with prior knowledge or previous studies on metrics such as number of fiber bundles for *in vivo* imaging, more detailed analysis of white matter structure requires a gold standard obtained with techniques such as optical imaging or electron microscopy.

In the digital phantom, local reconstruction of evenly sampled spiral data outperformed unevenly sampled spiral data. In human data, for instance, we found that SNR of images obtained from unevenly sampled spiral data were lower than for the other techniques. From simulation and *in vivo* experiments, we found that no additional advantageous exist in using unevenly sampled spiral data (Figure 1B) compared to evenly sampled spiral data.

The influence of sample density on denoising and on the accuracy of reconstruction of the FOD function using our sampling scheme is yet to be explored fully. Too few sampling points in the diffusion gradient direction domain ultimately limit the ability to capture signal periodicity. Fourier-based low-pass filtering in gradient direction domain enables the interpretation of the signal from spirally sampled data in terms of a series of Fourier coefficients. This provides the opportunity for future studies to explore interpolation in the diffusion gradient direction domain as a method to decrease the number of samples, and consequently, the acquisition time without a corresponding trade-off in accuracy of fiber reconstruction.

Features embedded in the acquired signal shape such as the value and location of signal extrema in each voxel may contain biologically relevant information, which can be explored to obtain data-driven metrics from the lop-DWI results. Low over-estimation rates with the use of the lop-DWI method can potentially improve tractography results, i.e., less false positive tracts (44). Furthermore, filtering in the spatial domain can be combined with lop-DWI to improve the signal further. In addition, continuity in the acquisition of spiral sampling scheme provides the possibility to apply techniques for smoothing continuous data such as dynamic non-local means based denoising (45). Our scheme cannot be applied to existing evenly sampled HARDI datasets. To apply the method to DWI data already acquired using even sampling schemes, an interesting study would be to find an optimum arrangement of acquired images from evenly sampled datasets to form a pseudo-spiral scheme.

## Conclusion

A new pre-processing method for DWI data based on an evenly distributed spiral sampling scheme in the gradient direction domain has been evaluated. The evenly distributed spiral sampling scheme results in voxel signals that concentrate to the low-frequency range of the Fourier spectrum. This knowledge about signal formation was exploited for denoising diffusion-weighted images at voxel level. The method was quantitatively validated on phantom and human data, and improvements in SNR and local reconstruction were observed. The evenly distributed spiral sampling scheme provides a new way to investigate patterns in sets of diffusion-weighted images at the voxel level of biological relevance.

## Conflict of Interest Statement

The authors declare that the research was conducted in the absence of any commercial or financial relationships that could be construed as a potential conflict of interest.
